# Hypomagnesemia-Induced Seizures Post Severe Acute Kidney Injury

**DOI:** 10.7759/cureus.26142

**Published:** 2022-06-21

**Authors:** Anwar AL-Omairi, Ahmed Alfarsi

**Affiliations:** 1 Child Health, Sultan Qaboos University Hospital, Muscat, OMN; 2 Pediatrics, Rustaq General Hospital, Rustaq, OMN

**Keywords:** acute kidney injury, pediatric seizure, tubulopathy, hypomagnesemia, acute tubular necrosis

## Abstract

Acute kidney injury (AKI) is a common complication in children admitted to pediatric intensive care units. It is known to be associated with increased morbidity and mortality. Here, we report prolonged, transient, increased renal magnesium (Mg) wasting after severe acute kidney injury presenting with a generalized tonic-clonic seizure.

## Introduction

Magnesium (Mg) is the second most abundant cation in the intracellular compartment. Mg is essential for many vital activities in the body, including DNA synthesis, generation of ATP, regulation of serum glucose, and neuromuscular stability [1-3]. Serum Mg level is tightly regulated by the kidney and intestine. Hypomagnesemia is relatively common in critically sick patients, however, it is sometimes overlooked, as it is commonly asymptomatic until the Mg level is critically low, which is seldom in clinical practice [4]. Hypomagnesemia is usually transient in the context of critical illness affecting the intake, absorption, or increase of renal wasting. Less commonly, hypomagnesemia is caused by a genetic mutation resulting in a defect in one of the mechanisms responsible for Mg handling by the kidney. The genetic disorders of Mg handling are complex and many of them are associated with other electrolytes imbalance and extra-renal symptoms [5-6]. Here, we report a case of severe hypomagnesemia provoked by a generalized tonic-clonic seizure in a three-month-old infant who was admitted with hypovolemic shock and severe acute kidney injury (AKI).

## Case presentation

A three-month-old girl with no significant perinatal history presented with vomiting, diarrhea, decreased activity, and poor feeding. At presentation, she was sick looking and clinically in compensated hypovolemic shock. She was tachycardic with a heart rate of 150 beats per min and blood pressure of 110/60 mmHg. She had signs of poor peripheral perfusion with cold peripheries. Her capillary refill time was 4-5 seconds. She was crying with no tears and her anterior fontanel was depressed. Initial lab work showed urea of 66.54 mmol/L, sodium of 112 mmol/L, potassium of 8.43 mmol/L, creatinine of 113 umol/L, and magnesium of 1.36 mmol/L (Table 1). She had a normal full blood count with no evidence of hemolysis. She was managed with normal saline boluses, sodium bicarbonate, and salbutamol nebulization in three doses. Then, after restoring the intravascular volume, she was kept on insensible loss and ongoing losses replacement volume to volume due to the concern of severe volume overload in the presence of anuric acute kidney injury (AKI). She was at the edge of requiring renal replacement therapy, however, she started making urine within a few hours, and in less than 24 hours, she went into the polyuria phase of AKI recovery with urine output exceeding 5 ml/kg/hour. Her serum sodium was gradually brought up to normal within 48 hours. On the third day, she had a generalized tonic-clonic seizure. Repeated extended electrolytes revealed very low Mg of 0.41 mmol/L associated with high urinary fractional excretion of Mg 8.82%. She was treated with IV Mg followed by an oral Mg supplement. Head ultrasound (US) was normal. The trial of discontinuing the supplement within three days resulted in another seizure with an Mg level of 0.47 mmol/L. She had required an Mg supplement for three weeks. After discontinuing the Mg supplement, her Mg level remained within normal and she remained seizure-free with no concerns regarding her growth and development.

**Table 1:** Investigation PCO2: partial pressure of carbon dioxide; HCO3: bicarbonate; US: ultrasound

Test	At presentation	Day 3	Normal reference range
Potassium (mmol/L)	8.43	3.25	(3.6 to 5.3)
Sodium (mmol/L)	112	135	(135 to 145)
Creatinine (umol/L)	113	13.86	(14 to 34)
Urea (mmol/L)	66.54	1.12	(2.8 to 8.1)
Calcium (mmol/L)	2.87	1.94	(2.15 to 2.55)
pH	6.9	-	(7.35 to 7.45)
PCO2 (mmHg)	25	-	(32 to 45)
HCO3 mmol/l	5	21	(21.8 to 26.9)
Albumin (g/L)	45 g/L	26.24	(38 to 54)
Ca albumin corrected (mmol/l	2.75	2.22	(2.15 to 5.55)
Phosphate (mmol/L)	2.50	1.19	(1.2 to 2.10)
Magnesium (mmol/L)	1.36 mmol/L	0.40	(0.69 to 0.91)
Hemoglobin (g/dL)	10.49 g/dL	9.36	(10.5 to 13.5)
Platelet (x10^9 ^/L)	755	379	(150 to 450)
Kidney’s ultrasound	Normal		
US head	Normal		

## Discussion

Mg is an essential cation to maintain cell hemostasis. In humans, the magnesium level is tightly regulated by balanced intestinal absorption and renal excretion [7]. The prevalence of hypomagnesemia in critically sick children is estimated between 47% and 60%. Hypomagnesemia is associated with increased morbidity, mortality, and length of stay [8-9]. Low magnesium level is one of the causes of seizures in children and adults [10-12]. The mechanism is likely related to a decrease in the inhibitory effect of magnesium on the N-methyl-D-aspartate (NMDA) receptor [12]. Causes of hypomagnesemia can be broadly divided into renal and digestive system causes. After taking a detailed history and physical exam, evaluating for renal wasting of Mg is a useful tool, to begin with. This can be done by calculating the fractional excretion of Mg on timed urine and serum samples. Having a fractional excretion of magnesium of more than 4% indicates significant renal Mg wasting [5]. Renal causes of hypomagnesemia are many. It can be transient due to tubular injury, hyperfiltration, or drug-induced. Cyclosporine, tacrolimus, thiazide, and proton pump inhibitors are known causes of hypomagnesemia [6]. Prolonged renal wasting of Mg warrants looking for genetic causes. With the recent advancement in genetic testing and increasing availability of testing, full gene panels are now available for different categories of kidney diseases. The hypomagnesemia genetic panel includes testing for mutation in CLDN 16, CLDN 19, CASR, SLC12A3, BSND, KCNJ10, FXYD2, HNF1B, PCBD1, MT-T1, SARS2, TRPM 6, EGF, EGFR, CNNM2, KCNA1, and FAM111A [5-6]. Mutation in many of these genes is associated with other electrolyte imbalances and extra-renal symptoms. Performing genetic testing is a standard of care when a genetic cause is suspected, as it doesn’t only confirm the diagnosis but extends to predicting extra-renal manifestations and helping with family planning. We deferred genetic testing for our patient, as her increased renal Mg wasting has resolved within three weeks.

AKI is known to be associated with increased morbidity, mortality, and prolonged length of stay [13-14], and there is some evidence postulating that an abnormal Mg level in AKI increases mortality and morbidity [15-18]. On admission, our patient had high serum magnesium along with stage III severe AKI by the Kidney Disease Improving Global Outcomes (KDIGO) classification. The likely explanation for the high initial magnesium level is the severally contracted intravascular volume and the decrease in glomerular filtration rate (GFR). Then, she had severe renal magnesium wasting, which had provoked a tonic-clonic seizure during the recovery phase of AKI. The low magnesium was likely secondary to the hyperfiltration and tubular injury that had been caused by the hypovolemic shock [5]. Hypomagnesemia, like many other electrolyte disturbances, is expected with severe AKI. Our patient had severe hyponatremia and hyperkalemia with metabolic acidosis. Severe hyponatremia is a well-known cause of seizures, however, our patient didn’t have any abnormal movement at presentation. Her sodium was brought up gradually, and it was corrected within 48 hours. At the time of the seizure, extended electrolytes were within the normal range apart from severely low serum Mg and borderline low potassium (Table 1). Thereafter, our patient had very isolated severe renal Mg. The recurrence of the seizure after stopping the Mg supplement proves this and having a low level during the second seizure proves the causality association between the seizure and the hypomagnesemia. The severity of hypomagnesemia in this patient was probably due to the small age of the infant and the immaturity of the renal tubule. Neonates are born with an immature renal tubule and low GFR; the kidney gradually matures till they reach adult GFR by the age of one year [19].

## Conclusions

Hypomagnesemia is a common condition in the pediatric intensive care unit. Hypomagnesemia per se is known to be associated with increased mortality and morbidity, and this effect probably increases when associated with AKI. Therefore, it is worth checking serum magnesium more frequently post AKI, especially in infants with severe polyuria, to avoid the neuromuscular and cardiac complications of hypomagnesemia.
